# Vick's Floral Guide for 1875

**Published:** 1875-03

**Authors:** 


*Vick's Floral Guide for* 1875.—As handsome a volume as any which have preceded it. Published by James Vick, Rochester, New York.

